# Development of Recombinase Polymerase Amplification Assays for Detection of *Orientia tsutsugamushi* or *Rickettsia typhi*

**DOI:** 10.1371/journal.pntd.0003884

**Published:** 2015-07-10

**Authors:** Chien-Chung Chao, Tatyana Belinskaya, Zhiwen Zhang, Wei-Mei Ching

**Affiliations:** 1 Viral and Rickettsial Diseases Department, Infectious Diseases Directorate, Naval Medical Research Center, Silver Spring, Maryland, United States of America; 2 Uniformed Services University of the Health Sciences, Bethesda, Maryland, United States of America; Aix-Marseille Université, FRANCE

## Abstract

Sensitive, specific and rapid diagnostic tests for the detection of *Orientia tsutsugamushi* (*O*. *tsutsugamushi*) and *Rickettsia typhi* (*R*. *typhi*), the causative agents of scrub typhus and murine typhus, respectively, are necessary to accurately and promptly diagnose patients and ensure that they receive proper treatment. Recombinase polymerase amplification (RPA) assays using a lateral flow test (RPA-nfo) and real-time fluorescent detection (RPA-exo) were developed targeting the 47-kDa gene of *O*. *tsutsugamushi* or 17 kDa gene of *R*. *typhi*. The RPA assay was capable of detecting *O*. *tsutsugamushi* or *R*. *typhi* at levels comparable to that of the quantitative PCR method. Both the RPA-nfo and RPA-exo methods performed similarly with regards to sensitivity when detecting the 17 kDa gene of *R*. *typhi*. On the contrary, RPA-exo performed better than RPA-nfo in detecting the 47 kDa gene of *O*. *tsutsugamushi*. The clinical performance of the *O*. *tsutsugamushi* RPA assay was evaluated using either human patient samples or infected mouse samples. Eight out of ten PCR confirmed positives were determined positive by RPA, and all PCR confirmed negative samples were negative by RPA. Similar results were obtained for *R*. *typhi* spiked patient sera. The assays were able to differentiate *O. tsutsugamushi* and *R. typhi* from other phylogenetically related bacteria as well as mouse and human DNA. Furthermore, the RPA-nfo reaction was completed in 20 minutes at 37^o^C followed by a 10 minute incubation at room temperature for development of an immunochromatographic strip. The RPA-exo reaction was completed in 20 minutes at 39^o^C. The implementation of a cross contamination proof cassette to detect the RPA-nfo fluorescent amplicons provided an alternative to regular lateral flow detection strips, which are more prone to cross contamination. The RPA assays provide a highly time-efficient, sensitive and specific alternative to other methods for diagnosing scrub typhus or murine typhus.

## Author Summary

Historically, rickettsial pathogens are among the leading causes of morbidity and mortality during military operations. Rickettsial diseases, lately, are reemerging in areas of known abundance or emerging in areas of unknown existence, posing a significant medical concern for local residents and travelers. The diseases are difficult to diagnose as they often share similar symptoms with many other diseases in the same geographical areas. Therefore, it is particularly challenging for clinicians to provide a timely and accurate diagnosis. A recombinase polymerase amplification (RPA)-based nucleic acid detection platform has been used to develop accurate, sensitive, specific, and easy-to-perform assays to detect *O*. *tsutsugamushi* or *R*. *typhi*, indicative of scrub typhus or murine typhus, respectively. These RPA assays provide similar limits of detection and specificity to that of qPCR. Unlike qPCR, they require no thermocycler and provide multiple end-point monitoring options amendable to different laboratory capabilities. This work presents an alternative assay platform for early detection of *O*. *tsutsugamushi* or *R*. *typhi* infection so that timely treatment can be prescribed in well-equipped laboratories as well as resource limited areas.

## Introduction

Rickettsial pathogens are among the leading causes of morbidity and mortality during military operations. In recent years, emerging rickettsial diseases have been reported throughout the world and are a significant medical concern for local and deployed personnel and travelers [1–3]. These pathogens include spotted fever group *Rickettsia* (SFGR, Far eastern spotted fever, Japanese spotted fever, Siberian and Queensland tick typhus, and Thai tick typhus just to name a few), typhus group *Rickettsia* (TGR, epidemic and murine typhus) and *Orientia tsutsugamushi* (scrub typhus, ST). Due to the high mortality rate of untreated rickettsial infections, early treatment with appropriate antibiotics is critical [4]. Doxycycline is the drug of choice, except in cases of pregnancy and tetracycline hypersensitivity. Symptoms of rickettsial infections are nonspecific and can be confused with a variety of other pathogens (e.g., dengue, malaria, leptospirosis) that require different treatment regimens. To ensure that appropriate treatment is initiated promptly, early diagnosis of rickettsial infections is critical.

Currently, the diagnosis of rickettsial diseases relies mainly on serological methods based on antibody detection [5, 6]. However, antibody based assays may not be adequate for the diagnosis of disease in the acute phase, as antibody levels may not be detectable at the onset of illness. Therefore, antigen/pathogen detection before the rise of antibody levels is important. Almost all antigen/pathogen detection assays for rickettsial diseases are based on polymerase chain reaction (PCR [7, 8]), quantitative real-time PCR (qPCR) or nested PCR targeting different genes, including 56 kDa [9], 47kDa [10], groEL [11] of *O*. *tsutsugamushi* and OmpB [7], 17 kDa [9], and gltA [12, 13] of *Rickettsia*. However, all these assays require end user training to operate the thermocycler, and a functional and well-calibrated thermocycler is difficult to obtain and maintain in resource poor settings. Recently, loop-mediated isothermal amplification has been developed [14–20] as a potential alternative for PCR based tests. In addition to groEL as the target [11], Huber et al. [21] demonstrated the use of the conserved 47 kDa gene as the target for the LAMP assay, which achieved similar sensitivity to that of qPCR.

Recombinase polymerase amplification (RPA) uses a mixture of prokaryotic recombinases to guide synthetic oligonucleotide primers to targets in the sample [22, www.twistdx.co.uk for detail description]. A strand displacing DNA polymerase (large fragment from *Bacillus subtilis* Pol I, *Bsu*) is used for primer extension [22]. The method is highly sequence specific in complex nucleic acid mixtures and in urine without the need for additional preparation of samples [23]. It offers amplification of the target sequence by reiterative oligonucleotide-primed DNA synthesis without the need to denature DNA at a high temperature. The assay can be performed between 24°C to 45°C [22] with very high efficiency so that detection of product from a single molecule is possible in 20 minutes [22]. The successful application of RPA is evident as shown in many recent publications. RT-RPA has been developed to detect HIV [24], Rift Valley Fever virus [25, 26], Ebola virus, Sudan virus and Marburg virus [26], MERS-CoV [27], foot-and-mouth disease virus [28], and Bovine Coronavirus [29]. Additionally, assays have been developed for detection of *Chlamydia trachomatis* in urine samples [23], diagnosis of *Cryptosporidiosis* in animal and patient specimens [30], and detection of *Neisseria gonorrhoeae*, *Salmonella enterica*, methicillin-resistant *Staphylococcus aureus* (MRSA) [31], *Francisella tularensis* [32], and Group B *Streptococci* [33]. Furthermore, the assay has been used in combination with ELISA for food analysis [34]. While RPA is similar to LAMP, in that no thermocycler is needed, the optimal reaction temperatures for RPA and LAMP are 37°C and 60°C, respectively. In addition, RPA is potentially easier to adapt to a multiplex format [22] than LAMP as RPA requires only one pair of primers rather than three like LAMP, in which the mixing of multiple pairs of primers may result in non-specific primer interactions and may limit the total amount of primer able to be added to the reaction. Finally, the incorporation of a probe can increase the specificity of the assay. This is particularly important, as the LAMP assay is known to result in non-specific amplification [35]. These advantages suggest that RPA may be a viable point-of-care nucleic acid detection method that can be utilized in resources-limited areas where these diseases are endemic.

In this report, we demonstrated that a lateral flow end point detection RPA (TwistAmp-nfo kit, hereafter RPA-nfo) could be performed at 37°C in 20 minutes to detect DNA from either *O*. *tsutsugamushi* or *R*. *typhi*. Similarly, a real-time fluorescent signal detection (TwistAmp-exo kit, hereafter RPA-exo) could be performed at 39°C to detect DNA from either *O*. *tsutsugamushi* or *R*. *typhi*. A detection limit similar to that of the gold standard qPCR was achieved by both RPA-exo and RPA-nfo. The specificity was evaluated using genomic DNA from closely related microorganisms at 1000 folds excess in copy number. The detection limit was established using a range of target genomic DNA from as high as 500 copies per reaction to less than 10 copies per reaction. Further evaluation was done using DNA extracted from liver, lung and spleen collected from *O*. *tsutsugamushi* infected mice, DNA extracted from clinical samples obtained from confirmed ST patient sera, or *R*. *typhi*-spiked normal human plasma. A cross contamination proof (XCP) lateral flow cassette was used to detect RPA-nfo amplicons. This method eliminates the need to open the reaction tube at the end of the reaction, thus minimizing the chance of contamination.

## Materials and Methods

### Ethics statement

The animal study protocol (#D11-06) was reviewed and approved by the Naval Medical Research Center Institutional Animal Care and Use Committee (IACUC) in compliance with all applicable Federal regulations governing the protection of animals in research. The experiments reported herein were conducted in compliance with the Animal Welfare Act and in accordance with the principles set forth in the “Guide for the Care and Use of Laboratory Animals,” Institute of Laboratory Animals Resources, National Research Council, National Academy Press, 1996.

### Design and synthesis of primers and probes

Oligonucleotide primers used for the RPA assays were manually designed based on the 47 kDa gene sequence from the Karp strain of *O*. *tsutsugamushi* (47-RPA) and the 17 kDa gene sequence from the Wilmington strain of *R*. *typhi* (17-RPA) per recommendation by TwistDx (Cambridge, UK). A minimum of 5 sets of primers and probes for each target were designed for different end-point detection methods. All primers and probes were synthesized by Eurofins MWG Operon (Huntsville, AL) and Biosearch Technologies (Petaluma, CA), respectively. The probes were HPLC purified.

### Experimental design

**Fig 1:**
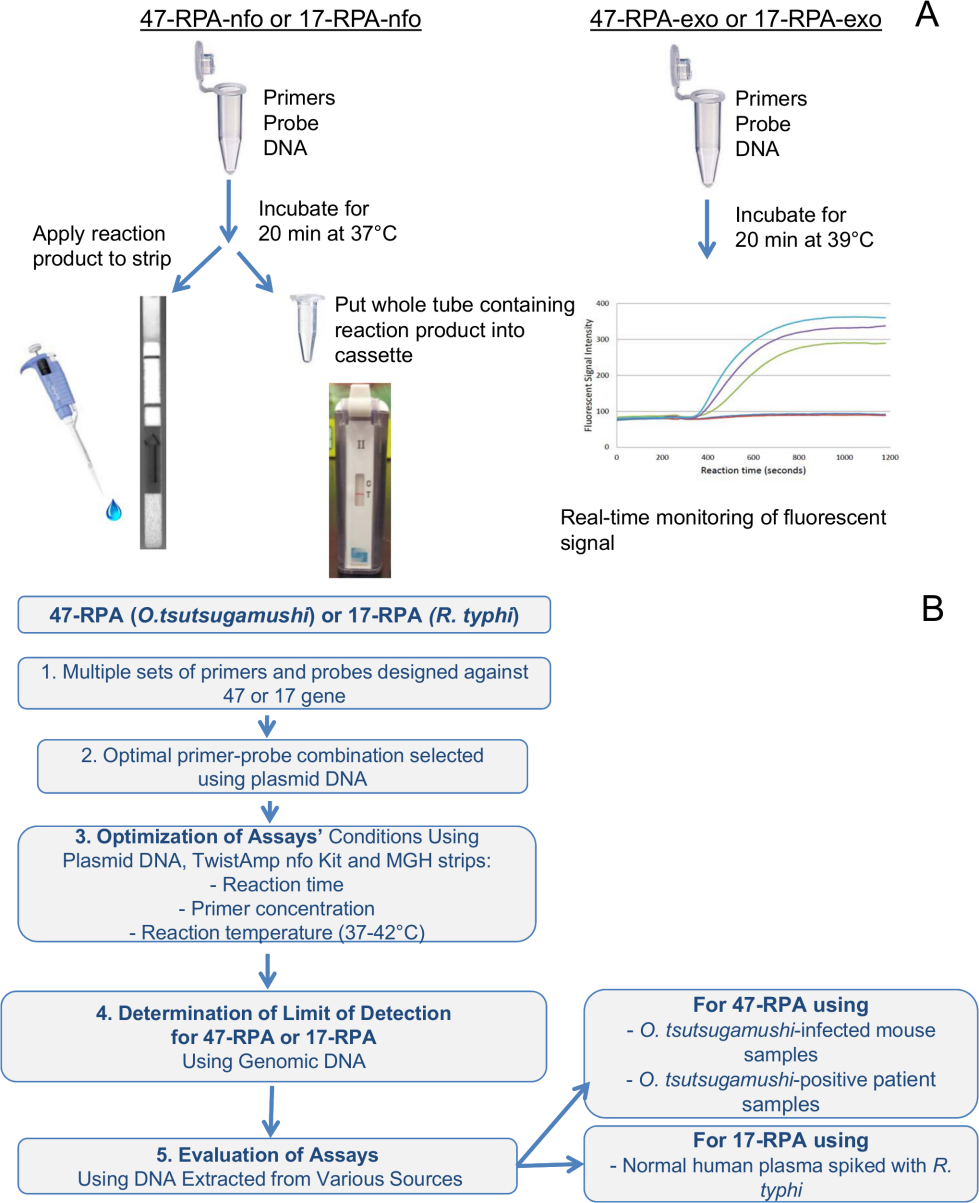
Experimental design. (A), the two TwistAmp kits used for detection of amplicons using a lateral flow strip, a cross contaminated proof (XCP) cassette, or a real-time monitoring of fluorescent signal. (B),a flowchart of the experimental design from evaluation of primers, optimization of assay conditions, determination of detection limit and evaluation of assay performance.

RPA-nfo and RPA-exo kits were both purchased from TwistDx (Cambridge, UK, www.twistdx.co.uk). The amplicons generated by the RPA-nfo were detected using a lateral flow strip or cassette (Fig 1A). RPA-exo was used to monitor fluorescent signals in real time using a fluorimeter (Fig 1A). Fig 1B shows a general scheme describing the design of the experiments.

### Preparation of plasmid and genomic DNA

PCR product of the 47 kDa gene sequence of *O*.*tsutsugamushi* Karp strain and the 17 kDa gene from *R*. *typhi* Wilmington strain was cloned into a VR1012 vector and pUC vector, respectively, following standard molecular biology technology. The plasmid was extracted from a 3 ml culture using the Qiagen plasmid mini kit (Qiagen, CA) following the manufacturer’s instruction. The concentration was determined using a Nanodrop (Thermo Fisher Scientific, CA) and the expected copy number of the target gene was calculated based on the size of the plasmid (http://cels.uri.edu/gsc/cndna.html). The purified plasmid was used as a template to optimize the RPA assays. Genomic DNA from multiple strains of *O*. *tsutsugamushi*, including Karp, AFSC4, AFSC7, Garton, Ikeda, and Boryong, TGR and SFGR was extracted from renografin gradient purified organisms using QIAamp Mini DNA kit (Qiagen, CA) as previously described [36]. Similarly, DNA from cultured *Leptospira*, *Coxiella burnetii*, and *Bartonella bacillifomis* was extracted by the same method.

### Extraction of DNA from ST confirmed patient blood, from mice infected by *O*. *tsutsugamushi*, and from normal human plasma (NHP) spiked with cultured *R*. *typhi*

DNA extracted from blood of patients with confirmed ST was provided to us by Dr. Yupin Suputtamongkol of Siriraj Hospital, Mahidol University, Bangkok, Thailand. The animal protocol to perform the mouse experiments (IDD-11-06) was approved by NMRC IACUC. According to the approved protocol, mice were challenged intraperitoneally (IP) by the Karp strain of *O*. *tsutsugamushi* and observed for up to 21 days. At the indicated time post infection, mice were sacrificed, and various organs were collected which included the lungs, liver and spleen. Genomic DNA was extracted from these organs following the manufacturer’s instructions of the QIAamp Blood and Tissue Mini DNA kit (Qiagen, CA). To prepare cultured *R*. *typhi*-spiked NHP, the number of *R*. *typhi* present in each of the three independently prepared *R*. *typhi* cultures was determined. To do this, DNA was extracted from 100 μl each of the three *R*. *typhi* cultures using the QIAamp Blood and Tissue DNA kit following the manufacturer’s instructions. The bacterial load was determined by qPCR to be within the range of 10^10^ -10^11^copies/ml. An initial dilution was made by adding cultured *R*. *typhi* into NHP at a 1:100 dilution followed by a serial dilution of 1:10 using NHP as a diluent to ensure that the final concentration of the 200 μl of spiked NHP was 2,000 copies/ml of cultured *R*. *typhi*. The DNA from each 200 μl *R*. *typhi*-spiked NHP was extracted using the QIAamp Blood and Tissue DNA kit. The DNA was used as template for the 17-RPA assay to evaluate its performance and qPCR was used to quantitate the copy number of the 17 kDa gene.

### Evaluation of different primer combinations

Each forward primer was mixed with different reverse primers separately to examine which pair performed the best using plasmid DNA (10–1000 copies) as template. Different reaction times, concentrations of primers and probes, and temperatures ranging from 37°C to 42°C were evaluated to select the most sensitive combination for the RPA reactions as per the manufacturer’s recommendations (TwistDx, Cambridge, UK). Each combination of parameters was performed at least twice. The temperature range was varied every 0.5 degrees within the range recommended by the manufacturer. The evaluation was done using the RPA-nfo for the RPA reaction and the results were evaluated using Milenia Genline Hybridetect-1 (MGH) strips by Millenia Biotec GmbH (Gieben, Germany).

### Recombinase Polymerase Amplification

Based on the evaluation performed in previous section, a final condition to carry out the recombinase polymerase amplification is described below. The reaction mixture was 50 μl using the RPA-exo or RPA-nfo. The reaction mixture contained rehydration buffer, recombinases, and *Bsu* strand displacing polymerase with the addition of 420 nM each of the forward and reverse primers, 120 nM of the FAM-tagged probe, at a total volume of 42.5 μl. After mixing these components, 2.5 μl of 280 mM magnesium acetate was pipetted into the tube lids, and 5 μl of DNA was added to the reaction mixture unless otherwise indicated. The lids were closed and the magnesium acetate was spun down into the reaction mixture to initiate the reaction. For RPA-exo, the tubes were placed into an ESEQuant tube scanner device (Qiagen, CA) and the reaction was conducted at 39°C for 20 minutes. The tubes were briefly agitated at 4 minutes after the initiation of the reaction, spun down and placed back into the tube scanner. The reaction in each tube was monitored in real time following the increase of fluorescent signals. For RPA-nfo, tubes were placed into a heating block at 37°C for 20 minutes. The tubes were briefly agitated at 4 minutes after the reaction was initiated, spun down and placed back into the heating block. The MGH strips were used to evaluate results according to the manufacturer’s instruction after the reaction was completed. For each reaction, 1 μl of sample amplicon was used for strip development. As an alternative detection method, after the reaction was completed, the entire reaction tube was inserted into the XCP cassette (BioUstar, Hongzhou, China) and the cassette was closed per the manufacturer’s instruction. The presence of a signal at the T line was an indication of positive detection of DNA using a 5’ FAM labeled probe.

### Determination of RPA-exo and RPA-nfo detection limits using genomic DNA

The copy number of genomic DNA extracted as previously described was determined by qPCR (below). The DNA was diluted to the desired copy number in a total volume of 5 μl to perform the RPA as described previously. The reaction was performed a minimum of 6 times for each amount of genomic DNA.

### Evaluation of 47-RPA using patient samples and DNA extracted from *O*. *tsutsugamushi* infected mice

Due to the limited availability of extracted DNA from scrub typhus confirmed patients, only 47-RPA-nfo was performed with 1 μL of DNA. For the DNA from *O*. *tsutsugamushi-*infected mice, the DNA was extracted from liver, spleen and lungs of these mice as described previously. The evaluation of 47-RPA-exo and 47-RPA-nfo was performed as described above using 5 μl of extracted DNA.

### Quantitative PCR

Quantitative PCR was performed to compare and confirm the detection limit of the RPA assay. Whenever the copy number of a sample is provided, it was obtained via qPCR using a standard curve built with a plasmid of known copy number. The 7500 Fast Real-time PCR System (Applied Biosystems, Foster City, CA) was used to perform qPCR reactions and analyze the results. The primers used here are the same as those previously used for amplifying the 47kDa gene sequence of the Karp strain of *Orientia* and 17 kDa gene sequence of *R*. *typhi* [10, 37]. A total reaction mixture of 20 μl contained 1 μM each of forward primer and reverse primer targeting the 47kDa gene of *Orientia* or 17 kDa gene of *R*. *typhi*, 1X RT^2^ SYBR Green qPCR Mastermix (SABiosciences, Frederick, MD), and DNA template. An initial 5-minute activation step at 95°C was followed by 40 cycles of 10 seconds at 95°C, 30 seconds at 60°C, and a melting curve determination cycle. To determine the amount of mouse genomic DNA in the total DNA extracted from organs of *O*. *tsutsugamushi* infected mice, qPCR was performed as described by Sunyakumthorn et al [38].

## Results

### Identification of the best combination of primers and probe

After running the RPA-nfo reaction with different primer and probe combinations, incubation temperatures and reaction time as mentioned in Materials and Methods, we applied several performance factors including total signal strength, absence of background signal on the MGH strip and detection limit to determine which combination of primer and probe, including concentration, reaction temperature and reaction time provided the lowest limit of detection. We used qPCR as a gold standard to quantitate the DNA to establish the detection limit. A primer and probe set that provided the best detection limit for the individual detection of *O*. *tsutsugamushi* or *R*. *typhi* DNA was identified (Table 1).

**Table 1:** Primer sequences for qPCR and RPA detection of *O*. *tsutsugamushi* and *R*. *typhi* DNA.

Pathogens	Method	Primer/probe name*	Primer sequence
*O. tsutsugamushi*	qPCR	47-F	AACTGATTTTATTCAAACTAATGCTGCT
		47-Rn	TATGCCTGAGTAAGATACGTGAATGGAATT
	RPA	47-7F	TAAAGTTGCATGATGGTTCAGAACTCATAGCA
		47-3R-nfo	Biotin-TATTGCAATAACCTGATCTCCTACTCTAGA
		47-3R-exo	TATTGCAATAACCTGATCTCCTACTCTAGA
		47probe12-nfo	5’d FAM-ATTAAAAATTAATTCTCCAGCAGCATTATCTTA-[THF]-GCGACTTTTGGCGACTCA-3’ blocker
		47probe12-exo	5’d ATTAAAAATTAATTCTCCAGCAGCATTATCTTA-[FAM-dT]-GC-[THF]-AC-[BHQ-1-dT]-TTTGGCGACTCA-3’ blocker
*R. typhi*	qPCR	PCR-17-F	AGTAGGTGTAGGYGCATTACTT
		PCR-17-R	GAGTGTAYTCACGGCAATATTGA
	RPA	17kDa 1F	CTAGAACTAACATCACAAAGAGCTTTAGAATC
		17kDa2R-nfo	Biotin-CAATATTGACCTGTACTGTTCCTATAAGTTT
		17kDa2R-exo	CAATATTGACCTGTACTGTTCCTATAAGTTT
		17-Probe1-nfo	5’d FAM-TAGCGGTAGTAACATAGAATGGCGCAATCCAGA-[THF]-AATGGCAATCATGGTTA- 3’ blocker
		17-probe1-exo	5’d TAGCGGTAGTAACATAGAATGGCGCAA-[FAM-dT]-CC-[THF]-GA-[BHQ-1-dT]-AATGGCAATCATGGTT-3’ blocker

*. All RPA primers and probes are named with nfo or exo to be specifically used for the nfo or exo detection method, respectively. Primers that are used for both nfo and exo are named without distinction.

### Using a lateral flow test to detect RPA amplicons of *O*. *tsutsugamushi* (47-RPA-nfo) or *R*. *typhi* (17-RPA-nfo) DNA provided a similar level of detection to that of qPCR

**Fig 2:**
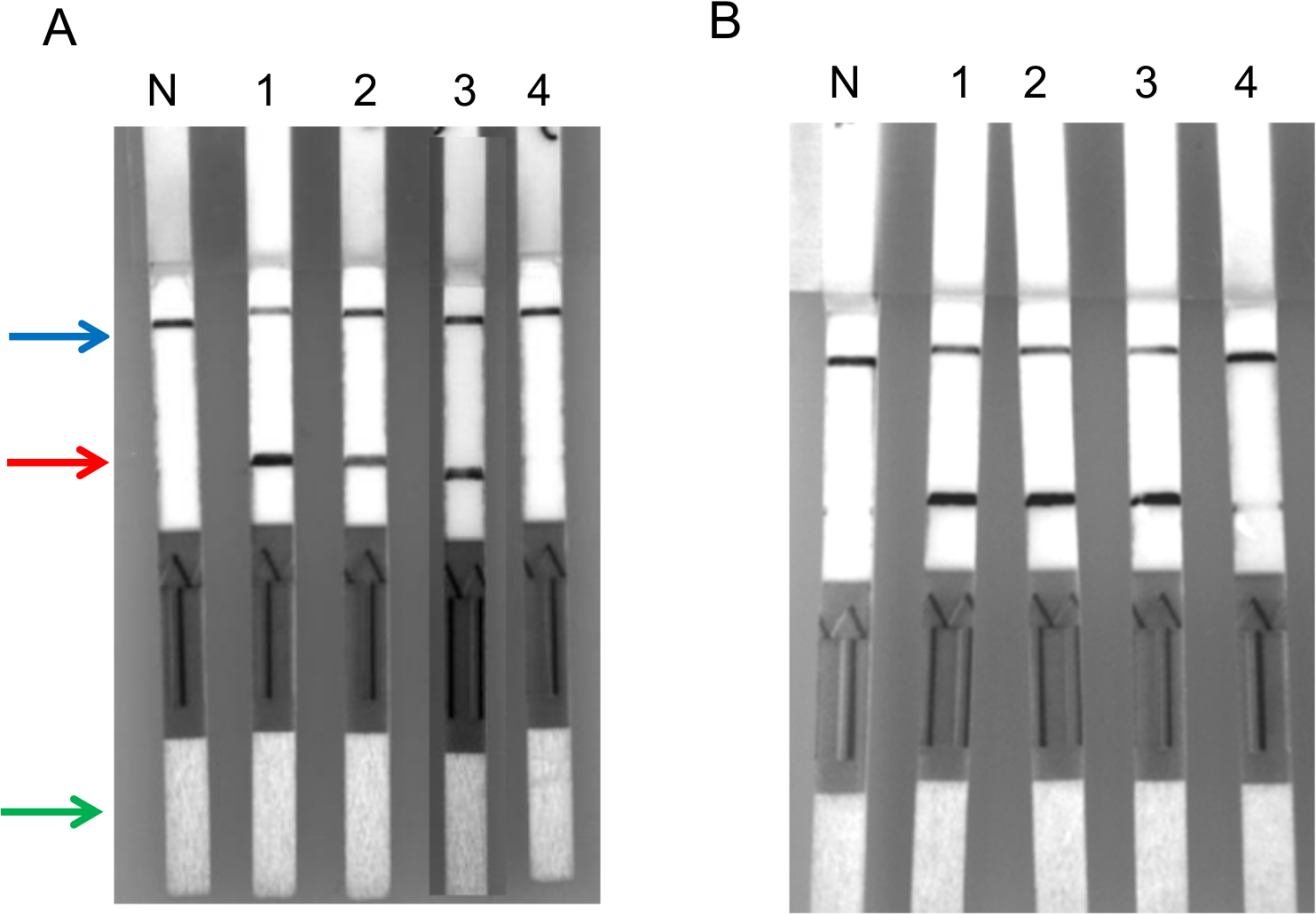
Representative results of RPA-nfo for the detection of *O*. *tsutsugamushi* or *R*. *typhi* genomic DNA. (A), different copy # of pure *O*. *tsutsugamushi* Karp strain genomic DNA as determined by qPCR were added in the RPA reaction. At the end of reaction, amplicons were removed and diluted as described in Materials and Methods for lateral flow strip detection of amplicons. Lane N: negative control, 1–4 contained 600, 220, 53 and 10 copies/reaction of *O*. *tsutsugamushi* DNA, respectively. (B), different copy # of pure *R*. *typhi* Wilmington strain genomic DNA as determined by qPCR were added as described. Lane N: negative control, lanes 1–4 contained 130, 45, 20, and 6 copies/reaction of *R*. *typhi* DNA, respectively. Control band, test band and sample application area are indicated by blue, red and green arrow, respectively.

Fig 2A shows typical results for the 47-RPA-nfo detection of different copy numbers of the 47 kDa gene. The 47-RPA-nfo appeared to be detecting 53 copies/reaction which is slightly higher than that by qPCR (10 copies/reaction). Different strains and isolates of *O*. *tsutsugamushi* including Karp, Kato, Gilliam, AFSC4, AFSC7, Ikeda, Garton, and Boryong were tested. While the region (156 bp) of the 47 kDa sequence that RPA primers targeted shared greater than 90% homology among these 8 strains (S1 Fig), the detection limit ranged from 10 to 400 copies per reaction. Similarly, the detection of *R*. *typhi* DNA using different copy numbers of the 17 kDa gene is representatively shown in Fig 2B. The 17-RPA-nfo was able to detect around 20 copies per reaction which is slightly higher than that by qPCR (6 copies per reaction).

### Fluorescent probe detection of RPA amplicons of *O*. *tsutsugamushi* (47-RPA-exo) or *R*. *typhi* (17-RPA-exo) DNA provided a similar level of detection to that of qPCR

**Fig 3:**
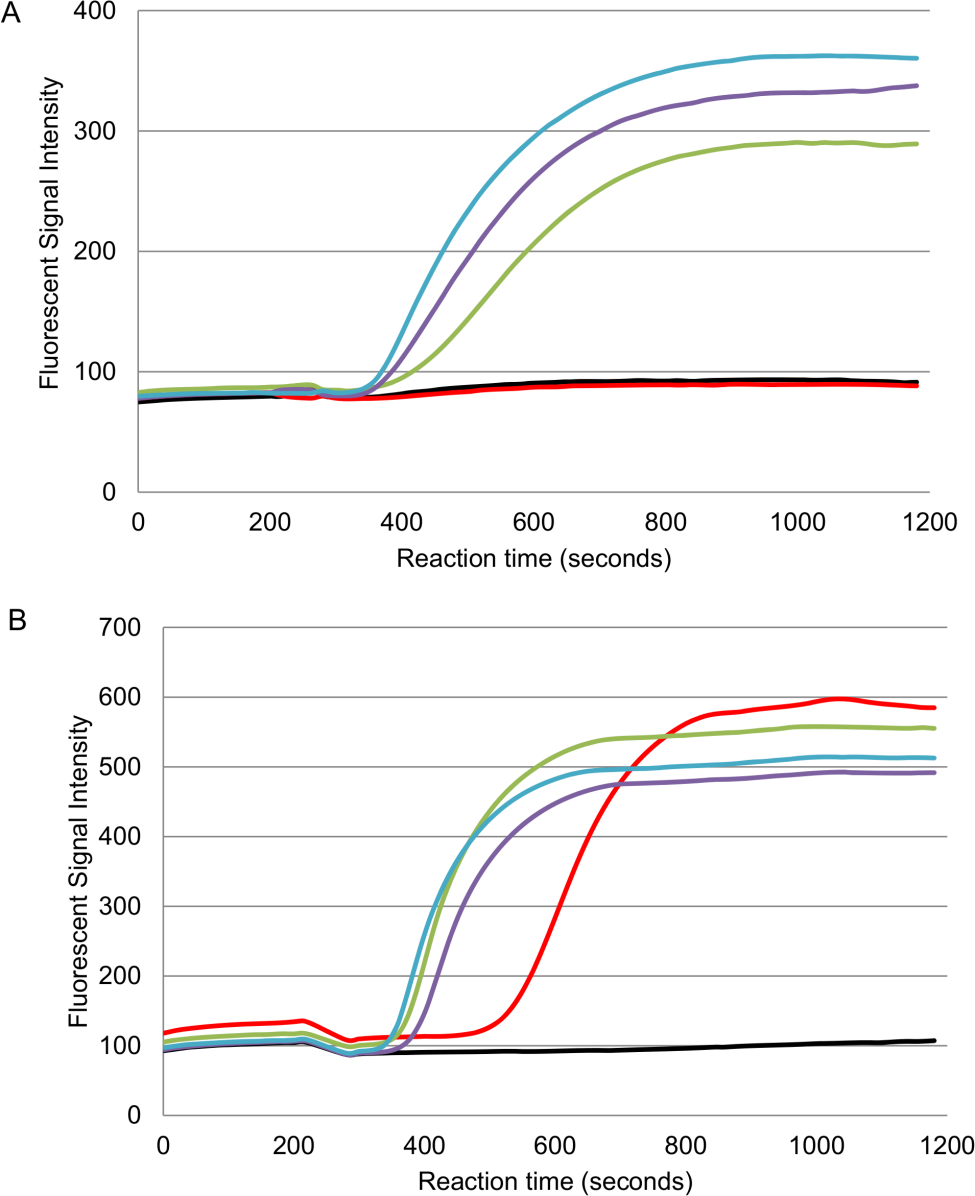
Representative results of RPA-exo for the detection of *O*. *tsutsugamushi* or *R*. *typhi* genomic DNA. (A), different copy # of pure *O*. *tsutsugamushi* Karp strain genomic DNA as determined by qPCR were added in the RPA reaction as described in Materials and Methods for real-time monitoring of amplicons. Negative control: (black line), 1000 (blue line), 200 (purple line), 50 (green line), and 10 (red line) copies per reaction of *O*. *tsutsugamushi* DNA, respectively. (B), different copy # of pure *R*. *typhi* Wilmington strain genomic DNA as determined by qPCR were added as described. Negative control: (black line), 500 (blue line), 250 (purple line), 125 (green line), and 50 (red line) copies per reaction of *R*. *typhi* DNA, respectively.

Results shown in Fig 3A indicate that the level of detection of the 47-RPA-exo was around 50 copies per reaction, this is similar to that of 47-RPA-nfo and qPCR at 53 and 10 copies per reaction, respectively. Similarly, the 17-RPA-exo showed a level of detection of 40 copies per reaction (Fig 3B) comparable to that of 17-RPA-nfo and qPCR, at 20 copies and 6 copies per reaction, respectively. Furthermore, the significant rise of fluorescent signal over time (i.e. slope) was observed no later than 10 minutes post initiation of the reaction, even though the signals plateaued within 20 minutes, suggesting the possibility of differentiating positive and negative samples based on the slope which reflects the change of fluorescent signal with time.

### Specificity of the RPA methods to detect only *O*. *tsutsugamushi* or *R*. *typhi* DNA

**Fig 4:**
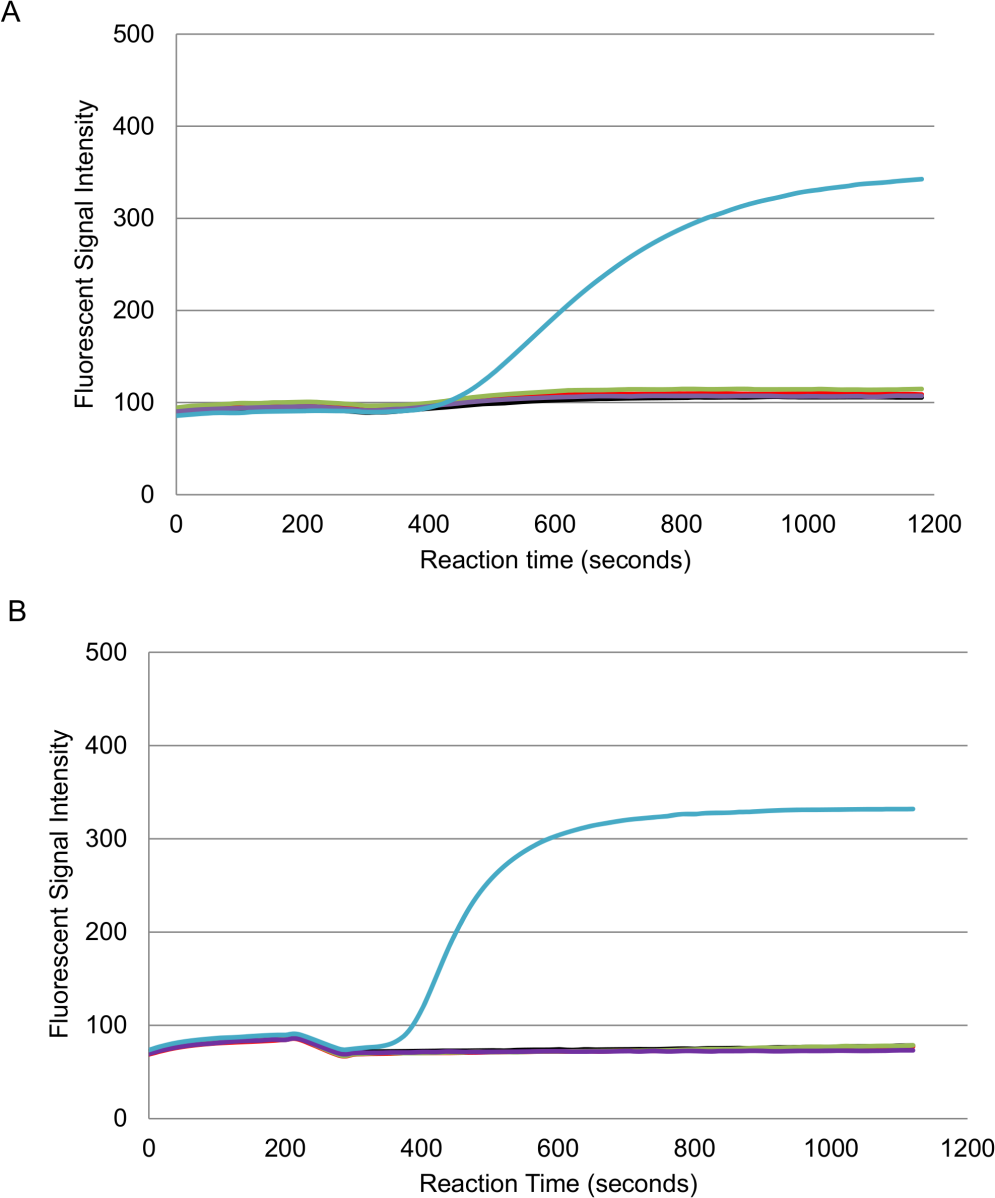
RPA-exo for the specific detection of *O*. *tsutsugamushi* or *R*. *typhi* DNA. The RPA-exo is specific for the detection of *O*. *tsutsugamushi* (A) or *R*. *typhi* (B) DNA even though non-target related DNA was present in 1000 folds more in copy # than the positive control DNA. Positive control (blue line) containing 200 or 350 copies per reaction of *O*. *tsutsugamushi* or *R*. *typhi* DNA, respectively, 5x10^4^ copies per reaction of *Coxiella burnetii* genomic DNA (green line), 1x10^4^ copies per reaction of *Leptospira* genomic DNA (purple line), and 6x10^4^ copies per reaction of *Bartonella* genomic DNA (red line), and negative control (black line).

To further evaluate whether the 47-RPA or 17-RPA only detected *O*. *tsutsugamushi* or *R*. *typhi*, respectively, purified genomic DNA from phylogenetically related organisms was tested. When DNA from *R*. *typhi*, *R*. *bellii*, *R*. *rickettsii*, *R*. *conorii*, *Leptospira*, *C*. *burnetii*, *B*. *bacilliformis* was used in the 47-RPA-exo, none were positive (Fig 4A). Consistent with this observation, the 17-RPA-exo did not show any reactivity with DNA from *O*. *tsutsugamushi*, *Leptospira*, *C*. *burnetii* or *B*. *bacilliformis* (Fig 4B). The same results were obtained using 47-RPA-nfo. It is noted that when 17-RPA-nfo was used to detect *R*. *conorii* and *R*. *rickettsii*, positive results were only observed when at least 10^4^ copies/reaction of DNA were present (S2 Fig), suggesting that the assay was much less sensitive to detect these two SFGR. Other SFGR, such as *R*. *honei* and *R japonica* were also evaluated to show that they were not detectable by 17-RPA-nfo unless 10^4^ or more copies of DNA were present, same results were obtained using 17-RPA-exo. Additionally, the performance of 17-RPA-exo was also evaluated using DNA extracted from *R*. *prowazekii*. The assay consistently detected as low as 80 copies per reaction. Taken together, these results are consistent with the notion that this 17-RPA is better suited to detect TGR.

### Detection limit of the RPA methods using extracted genomic DNA

Different amounts of extracted genomic DNA were used to evaluate the detection limit of the two RPA methods running at least 6 replicates for each assay. The 47-RPA-nfo and 47-RPA-exo had different detection limits whereas the 17-RPA-nfo and 17-RPA-exo had very similar detection limits. As shown in Table 2, when testing 47-RPA-exo, the assay detected 100%, 77% and 42% of samples with 100–120 copies, 40–60 copies and 10–12 copies per reaction, respectively. When 47-RPA-nfo was used, the assay was positive for 100%, 67%, 54% and 0% of samples with 500–550 copies, 200–250 copies, 100–120 copies and 40–60 copies per reaction, respectively. Based on these results, 47-RPA-exo had a significantly lower detection limit from that of 47-RPA-nfo. The 17- RPA-exo detected 88%, 91% and 43% of samples ranging from 100–120 copies, 40–60 copies and 5–15 copies per reaction, respectively. The 17-RPA-nfo, on the other hand, detected 100%, 73%, 71% and 57% of samples ranging from 100–120 copies, 40–60 copies, 20–25 copies and 5–15 copies per reaction, respectively. There was no statistical significant difference (Table 2, Fisher’s exact test) in percentage of positive samples detected for various ranges using 17-RPA-exo and 17-RPA-nfo, suggesting that the detection limit was comparable between 17-RPA-exo and 17-RPA-nfo.

**Table 2:** Evaluation of the detection limit of RPA-nfo and RPA-exo using genomic DNA^*^.

RPA method	Sample load^a^ (copies/reaction)	Percent positive
47- nfo	500–550	100
	200–250	66.7
	100–120	53.8^b^
	40–60	0^c^
47- exo	100–120	100^b^
	40–60	77^c^
	10–12	41.7
17- nfo	100–120	100
	40–60	72.7
	20–25	71.4
	5–15	57.1
17- exo	100–120	87.5
	40–60	90.9
	5–15	42.9

*. Percent positive is calculated based on [# of positive/total # of samples tested]. For each sample load condition, at least 6 independent replicates were analyzed.

^a.^ The number given in sample load represents the range of copy number of target gene per reaction used in RPA reactions as determined by qPCR.

^b.^ Significantly different as calculated by Fisher’s exact test (p = 0.0009) using GraphPad Software (http://www.graphpad.com/quickcalcs/contingency1/).

^c.^ Significantly different as calculated by Fisher’s exact test (p = 0.0012) using GraphPad Software (http://www.graphpad.com/quickcalcs/contingency1/).

### Evaluation of 47 kDa based RPA to detect *O*. *tsutsugamushi* DNA isolated from nested PCR confirmed patient blood samples and qPCR confirmed laboratory, *O*. *tsutsugamushi*-infected mouse samples

**Fig 5:**
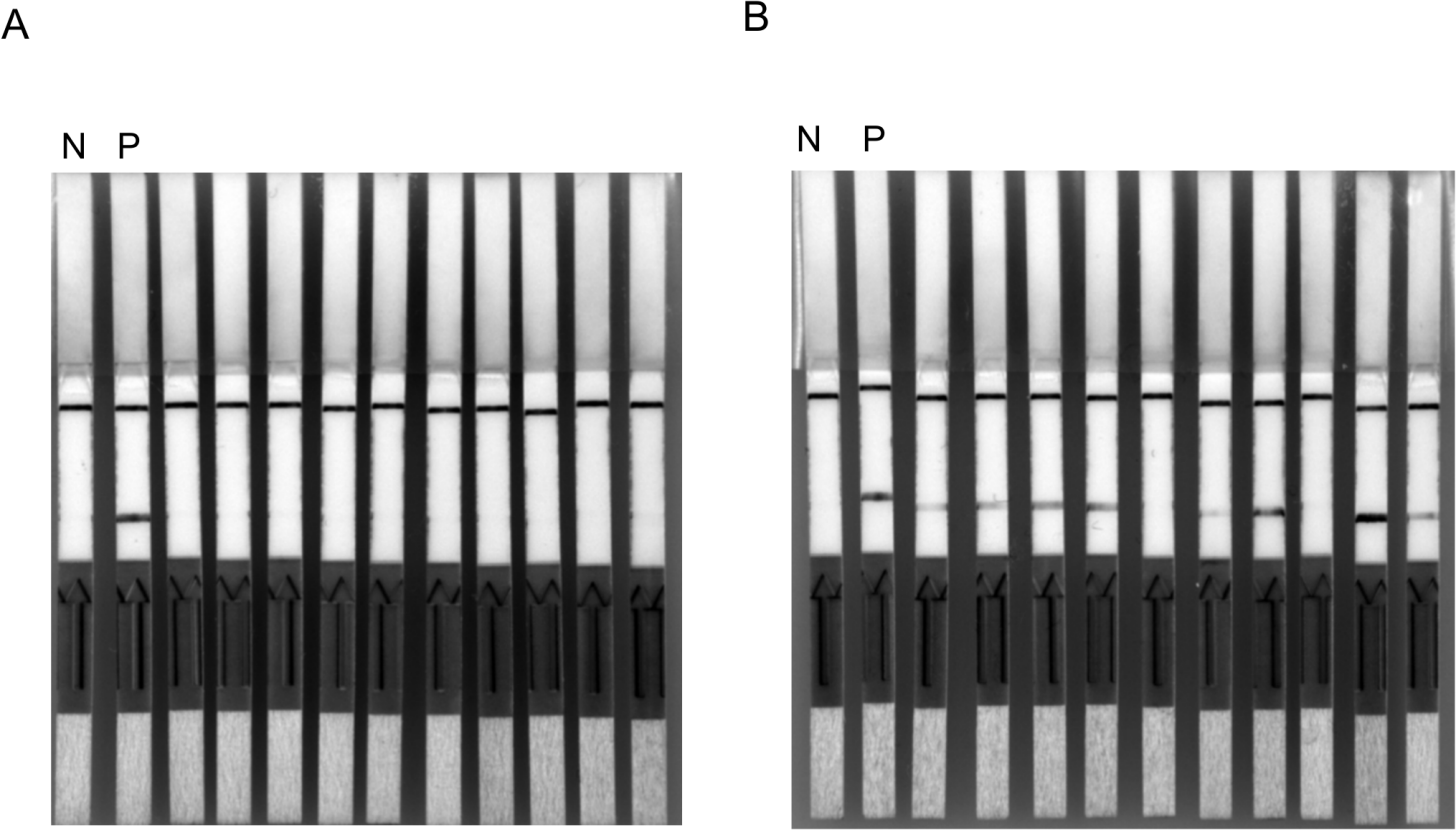
Evaluation of RPA-nfo detection of *O*. *tsutsugamushi* DNA in PCR confirmed positives and negatives. RPA-nfo reaction was performed as described in the Materials and Methods. (A), ten negative and (B), 10 positive samples (previously confirmed [19]) were evaluated along with positive (P) and negative (N) controls.

The 47-RPA was evaluated using a total of 10 positive and 10 negative human samples, the results showed that 8 out of 10 positives were 47-RPA-nfo positive while all 10 negatives were negative (Fig 5), similar to what was observed using the LAMP method [21]. Even though we only ran 47-RPA-nfo using 1 μl instead of 5 μl DNA due to the limited availability of extracted DNA, nevertheless, the limited number of clinically confirmed ST patients shows that 47-RPA exhibited 80% sensitivity and 100% specificity. The 47-RPA was also evaluated using extracted DNA from liver, spleen and lung from laboratory, *O*. *tsutsugamushi*-infected mice. These samples were confirmed qPCR negative before day 4 post infection and then a gradual increase in quantity was seen as shown in Table 3. The same extracted DNA was used as a template for both 47-RPA-exo and 47-RPA-nfo. Among these samples, 3 qPCR negative samples were determined negative by 47-RPA-exo and 47-RPA-nfo. Among the 9 qPCR positive samples, 7 of them were detected positive by 47-RPA-exo and 47-RPA-nfo. These results demonstrated that 78% sensitivity and 100% specificity was achieved using DNA extracted from organs obtained from *O*. *tsutsugamushi*-infected mice. Taken together, it is concluded that 47-RPA provides around 80% sensitivity and 100% specificity.

**Table 3:** RPA-nfo and RPA-exo detection of *O*. *tsutsugamushi* DNA extracted from liver, spleen and lung of infected mice.

Day post infection^a^	Organs	Copy number of 47 kDa gene^b^	RPA-nfo	RPA-exo
<4	Liver	ND^c^	(-)	(-)
	Spleen	ND	(-)	(-)
	lung	ND	(-)	(-)
4	Liver	3.3 + 0.9	(+)	(+)
	Spleen	4.3 + 0.7	(-)	(-)
	lung	0.5 + 0.3	(-)	(-)
7	Liver	(1.96 + 0.65)x10^3^	(+)	(+)
	Spleen	220 + 170	(+)	(+)
	lung	62.3 + 23.4	(+)	(+)
11	Liver	(6.2 + 2.9)x10^5^	(+)	(+)
	Spleen	(2.29 + 1.13)x10^4^	(+)	(+)
	lung	(2.37 + 0.32)x10^4^	(+)	(+)

^a^ Liver, spleen and lungs were collected at five time points before day 4 post infection. DNA from one mouse per time point was extracted as described in Materials and Methods and used for RPA reactions as described. Liver, spleen and lungs from five mice each at day 4, 7 and 11 post infection were collected. DNA was extracted as described to perform RPA reactions.

^b^ The copy number of *O*. *tsutsugamushi* 47 kDa gene was determined by qPCR (expressed as copy number per μL of DNA template) and presented as mean ± standard deviation of five mice. 5 μL of the DNA was used for RPA-exo or RPA-nfo detection.

^c^ ND: not detectable

### Evaluation of 17-RPA to detect *R*. *typhi* DNA isolated from normal human plasma spiked with cultured *R*. *typhi*

Due to the lack of qPCR confirmed *R*.*typhi* positive patient samples, the clinical performance of the 17-RPA was evaluated by spiking normal human plasma with cultured *R*. *typhi*. The results of the qPCR quantitation, 17-RPA-exo, and 17-RPA-nfo are shown in Table 4. All qPCR positive samples were positive by 17-RPA-exo and 17-RPA-nfo. Based on the amount of *R*. *typhi* DNA spiked into NHP and the final copies of *R*. *typhi* in the extracted DNA, the DNA extraction resulted in average DNA recovery of 81% (48%–104%).

**Table 4:** Extraction of *R*. *typhi* DNA from normal human plasma spiked with *R*. *typhi* for evaluation of RPA-exo and RPA-nfo.

*R*.*typhi* culture (spiked into normal human plasma)	Copy number of 17 kDa gene^a^	RPA-nfo	RPA-exo
E4	7.3±2.5	(+)	(+)
G20	8.3±1.4	(+)	(+)
L5A	3.8±1.6	(+)	(+)

^a^ The copy number of *R*. *typhi* 17 kDa gene was determined by qPCR (expressed as copy # per μL of DNA template) and presented as mean ± standard deviation of three replicates. 10 μL of the extracted DNA was used for RPA-exo or RPA-nfo detection. The expected copy number of 17 kDa gene present in each of the spiked sample was 8 copies per μL based on 2000 copies/mL of 200 μL of spiked NHP and assuming 100% recovery after DNA extraction.

### Using a commercially available XCP cassette to replace MGH strips

**Fig 6:**
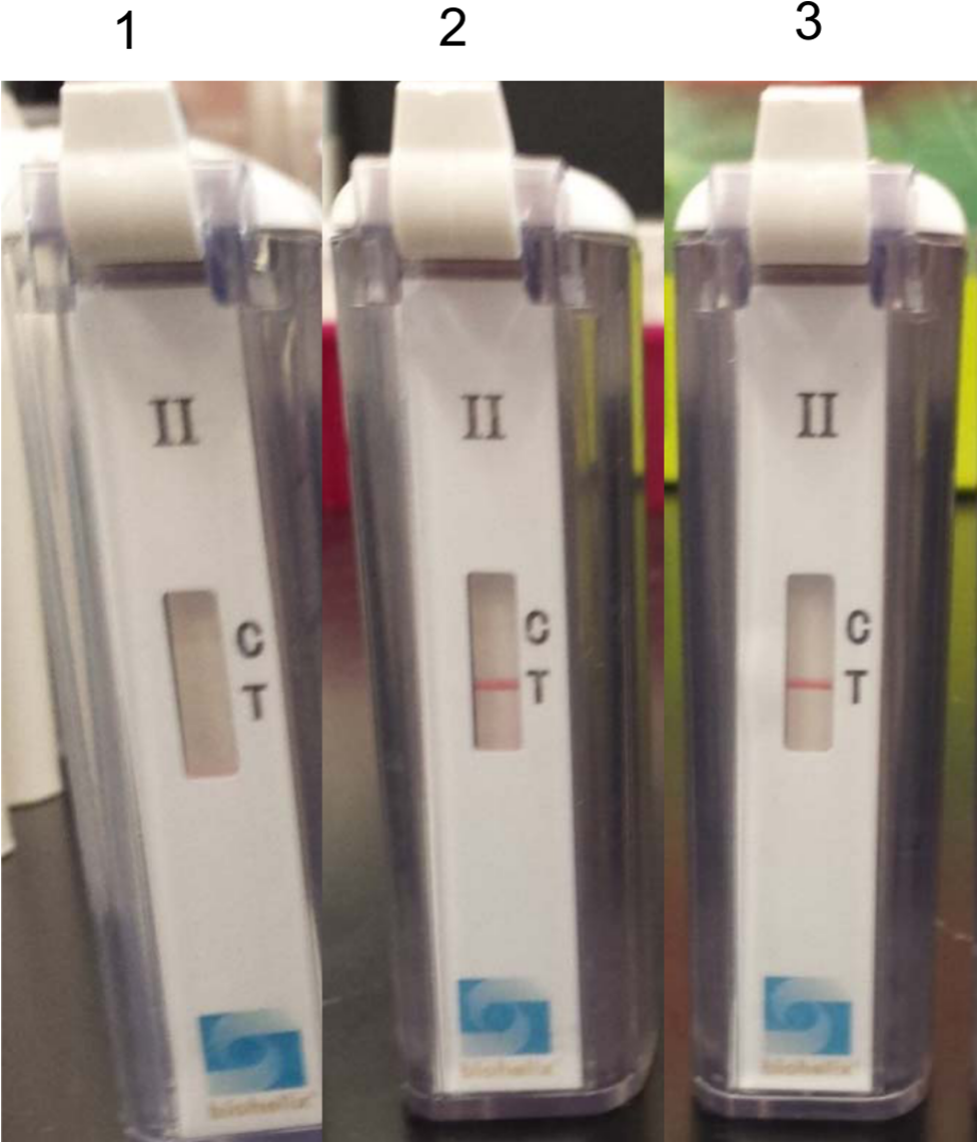
Evaluation of RPA-nfo assay using XCP cassette. The RPA-nfo assay for both 17 kDa gene of *R*. *typhi* and 47 kDa gene of *O*. *tsutsugamushi* was performed as described in Materials and Methods. Upon completion of the assay, the reaction tube was transferred to the cassette and the cassette was closed. The appearance of a red line along the T mark indicated the positive detection of FAM labeled probe. Cassette 1 reaction mixture contained no DNA template and complete primer sets for 47 kDa gene and 17 kDa gene with FAM labeled probes. Cassette 2 was the same as cassette 1 with the addition of 350 copies of *O*. *tsutsugamushi* DNA. Cassette 3 was the same as cassette 1 with the addition of 350 copies of *R*. *typhi* DNA. *Note: As mentioned previously, no C line was expected because there are no DIG-labeled primers in the RPA reactions, as these cassettes were initially designed for the LAMP assay.

Using the XCP cassette to replace the MGH strips at the end of the RPA-nfo reaction, the FAM labeled probes showed signal at the T line (Fig 6), just like the strips. These results demonstrated the potential usage of this XCP cassette as a replacement for the MGH strips for the detection of RPA-nfo amplicons. As the XCP cassette does not require opening the reaction tube and uses all of the reaction volume for detection, it is conceivable that the usage of the XCP cassette to detect the end product could be more sensitive, and present less opportunity for contamination than the usage of MGH strips to detect the RPA-nfo product.

## Discussion

The data presented here shows that RPA is a method that could be used to detect plasmid DNA or DNA extracted from patient samples, infected mice, and pure organisms with a detection limit of tens of copies, within 20 minutes. This is generally comparable to that of qPCR (Figs 1 and 2 and Table 3). This detection limit observed is not likely due to the limitation of the RPA assay as it has been shown to detect product amplified from a single molecule [22]. Rather, it is conceivable that the detection limit can be improved if different genes are selected for assay development. In addition to having a similar detection limit, the RPA method has several advantages over qPCR, making it an attractive alternative. Firstly, a heating block that is capable of maintaining a temperature of 37°–39°C for 20 minutes is sufficient to perform the reaction. Secondly, reaction mixtures are pre-made and provided by TwistDx. With the addition of water, template, primers and probe, the reaction is then initiated upon mixing, thus minimizing the potential for contamination often observed with other nucleic acid amplification methods. Thirdly, the method offers multiple end-point detection options with similar detection limits that could fit into many different laboratory settings, thus making one assay applicable in a well-equipped laboratory, a mobile laboratory or a rural area where instruments and infrastructure may be limited. Finally, successful detection of *O*. *tsutsugamushi* from clinical and mouse samples known to be infected, demonstrated the potential clinical application of the assay.

The RPA method was developed almost 10 years ago. However, it has not been accepted as well as some other isothermal amplification methods, i.e., LAMP. Although the method is relatively easy to perform with all the advantages described previously, one difficulty lies in the design of the primers and probes. There is no software available to assist in properly designing primer and probe sets. Furthermore, the requirement of extra length primers and probes has made the manual design more difficult than other methods, such as qPCR, PCR and LAMP which have well established software for primer and probe design. The relatively rare modifications on the probes also add cost to the synthesis. Consequently, it may prevent the evaluation of large numbers of potential primers and probes to lead to the most sensitive and specific assay possible. Nevertheless, the advantages seem to outweigh these minor drawbacks as we and many others [22–34] were able to demonstrate the utility of the assay for detection of closely related pathogens without sacrificing sensitivity or specificity. In addition to the development of RPA for detection of *O*. *tsutsugamushi* and *R*. *typhi*, the RPA-nfo method was developed *C*. *burnetii* DNA [39]. The detection limit of these assays was comparable to that of qPCR.

In the detection of *O*. *tsutsugamushi* using RPA methods, there appeared to be a slight difference in detection limit between RPA-nfo (54% of 100–120 copies per reaction) and RPA-exo (77% of 40–60 copies per reaction). On the contrary, the difference in detection limit was less pronounced for *R*. *typhi* detection as shown in Table 2. Due to the observations that RPA-nfo requires higher copies of target gene for positive detection in general, the RPA-nfo assays for both targets were evaluated using higher copies/reaction or additional ranges of copies/reaction to best estimate the detection limit of RPA-nfo assay. The test results using varying ranges of genomic DNA for 47-RPA and 17-RPA-demonstrated differences in percent positive detection, for most tested ranges, the assay provided around 50% positivity regardless of whether it was RPA-nfo or RPA-exo. Additionally, these results are consistent with the notion that lateral flow strips are generally regarded as not-as-sensitive as other detection methods, such as fluorescent signal detection. The use of strips to evaluate the results of RPA-nfo requires the reaction tubes to be opened to remove samples after the reaction is completed, thus adding extra post reaction time and increasing the possibility of cross contamination due to the presence of abundant amplicons. The cassette form of the lateral flow strips essentially eliminates cross contamination yet still requires additional time to obtain the final results. It is worth noting that both MGH strips and XCP cassettes are commercially available and they were used without any attempt to optimize the performance. Thus, it is conceivable that further optimization of these devices could improve the sensitivity and eliminate the difference in sensitivity between 47-RPA-nfo and 47-RPA-exo.

The target genes, 47 kDa and 17 kDa, were selected for *O*. *tsutsugamushi* and *R*. *typhi* DNA detection, respectively, based on their relatively conserved nature in sequence. The 47 kDa gene codes one of the major protein antigens recognized by sera from patients infected with many strains of *O*. *tsutsugamushi* [40]. The sequences of 47 kDa proteins have been compared to show greater than 96% identity [41] between strains with the exception of one recently identified strain, *O*. *chuto* [42], that shared only about 83% identity [41]. The gene has been used as the target for the development of PCR, qPCR, and LAMP assays and has shown consistent results in sensitivity, specificity, and broad reactivity toward many different strains of *O*. *tsutsugamushi* [10, 21]. While the region (156 bp) of 47 kDa that RPA targeted shared greater than 90% homology among the 8 strains tested (S1 Fig), it is noted that a different detection limit was observed between strains. In spite of the difference in detection limit observed for different strains, these detection limits still fall within the broad expected level of *O*.*tsutsugamushi* circulating in patients’ blood [43]. Since the level of *O*. *tsutsugamushi* present in patient’s blood is also associated with the disease severity, it is likely that the current 47-RPA-exo and 47-RPA–nfo need further improvement to achieve lower detection limits in order to be more clinically applicable.

Similarly, the 17 kDa gene has been used to confirm the presence of *Rickettsia* DNA in potentially infected samples [9, 37]. Additional targets were used to delineate whether the infection was due to TGR or SFGR [7, 8]. It is noted that the designed primers and probe for 17-RPA, which are located at the latter half of the 17 kDa sequence, showed a lower detection limit for *R*. *typhi* DNA while it required a copy number greater than 10^4^ of *R*. *conorii* and *R*. *rickettsii* DNA to be detectable by 17-RPA-nfo (S2 Fig). Same observation was made for *R*. *honei* and *R*. *japonica*. The 17 kDa genes from SFGR that were tested share 89% of the identity with the full length 17 kDa gene of *R*. *typhi*. The 17 kDa gene of *R*. *typhi*, shares 95% of the identity with the 17 kDa gene of *R*. *prowazekii*, another member of the TGR. The 17 kDa gene of *R*. *conorii*, *R*. *rickettsia*, *R honei*, an*d R*. *japonica* show no more than 87% of the identity with that of *R*. *typhi* within the region where the primers and probe are located (134 bp), the identity between *R*. *typhi* and *R*. *prowazekii* is 97%. Since the sensitivity is clearly different between *R*. *typhi* and the two species in the SFG *Rickettsia* using just one set of primers and probe, it suggests that 87% identity is not enough to use one set of primers and probe to detect both TG and SFG *Rickettsia* with equal sensitivity. Therefore, another set of primers and probe is needed for SFGR in order to have a similar detection limit to that for *R*. *typhi*. This difference in reactivity toward TGR and SFGR suggests that the assay is highly specific. While the detection limit using NHP spiked with cultured *R*. *typhi* was about 2000 copies/ml (Table 4), it is higher than the median of 210 DNA copies/mL in blood of confirmed murine typhus patients [44], suggesting that the assay may not be sensitive enough to be clinical useful. Since no evaluation of 17-RPA was done using clinical samples, it is warranted that additional evaluations should be done to further characterize the clinical sensitivity and specificity of the 17-RPA.

Both RPA-exo and RPA-nfo kits are specific only to the targeted organisms. This is demonstrated in Fig 3A and 3B, and Table 3. In Fig 3, it is shown there was no amplicon unless the target DNA (i.e., *O*. *tsutsugamushi* or *R*. *typhi*) was present even though the non-target DNA was present in 1000 folds excess compared to the target gene. The presence of excessive human DNA and mouse DNA apparently did not interfere with the detection of the target gene, suggesting that the assay provided sufficient specificity for clinically relevant samples. This is supported by the results showing that 8 out of 10 PCR confirmed *O*. *tsutsugamushi* positives were positive and 10 out of 10 PCR confirmed *O*. *tsutsugamushi* negatives were negative. While we achieved 80% sensitivity and 100% specificity using a limited number of clinical samples, we also evaluated the specificity and sensitivity of the 47-RPA-exo and–nfo more closely using samples from mice infected by live *O*. *tsutsugamushi*. As shown in Table 3, no samples collected prior to day four post infection had qPCR detectable *O*. *tsutsugamushi*. As expected due to the similarity in sensitivity between qPCR and RPA, none of these samples were positive by either 47-RPA-nfo or 47-RPA-exo. On the contrary, as the infection progressed, *O*. *tsutsugamushi* DNA became detectable by qPCR and 47-RPA as early as day four post infection in all organs evaluated, even though the number of *O*. *tsutsugamushi* detected was low. For those samples with low copy of *O*. *tsutsugamushi* DNA by qPCR, not all of them were 47-RPA detectable. Overall, both 47-RPA-exo and–nfo showed consistent results of 78% sensitivity and 100% specificity, similar to the observation made using DNA extracted from scrub typhus patients. It is noted that the number of *O*. *tsutsugamushi* was undetectable before day 4 post infection or the number was very low at day 4 post infection. This is probably related to the number of live organisms injected into the mice, the naturally-slow growth of *O*. *tsutsugamushi* and the route and rate of dissemination of *O*. *tsutsugamushi* once it enters into the mice. The amount of mouse DNA was not the same for all samples, so we normalized the copy number of *O*. *tsutsugamushi* to a mouse complement factor D gene [cfd, 38]. The ratio of mouse DNA to *O*. *tsutsugamushi* DNA ranged from as low as 1.9 (lung, day 11) to as high as 76800 (lung, day 4). These results suggested that the ratio of non-target DNA to target DNA did not affect the detection of target DNA with a 10^4^ dynamic range and that either 47-RPA-nfo or 47-RPA-exo has the potential applicability in clinical samples for diagnosis of infection. Nevertheless, the number of samples evaluated in this report is limited and the evaluation of additional clinical samples is needed to better describe the clinical sensitivity and specificity.

In this work, we took advantage of the similarity between LAMP and RPA-nfo in detecting FAM labeled amplicons, provided a reverse primer is labeled with biotin. The BioUStar XCP cassette was designed specifically for the detection of LAMP amplicons when a FAM labeled loop primer along with a biotin labeled reverse primer were used together in the reaction. In the original design, the C-line signal indicates an internal control containing a DIG labeled loop primer while the T-line signal indicates a positive sample containing a FAM labeled loop primer (www.bioustar.com). The results in Fig 6 clearly demonstrated that the assay was only positive at the T line when correspondingly labeled probe was present together with all primers and DNA templates. While it is true that the XCP cassette was not designed to detect RPA-nfo products, our approach confirmed the possibility of using the XCP cassette for RPA-nfo to provide an end point readout with no cross contamination. Furthermore, this provides a single XCP cassette that can be used for the detection of practically all FAM (or DIG) labeled probe amplicons, including qPCR, LAMP and RPA-nfo in a pick-and-choose manner to select those assays for target organisms that are most relevant to the local area where the assay will be performed.

In conclusion, the work presented here demonstrates the development of RPA-nfo and RPA-exo for the detection of *O*. *tsutsugamushi* or *R*. *typhi* DNA. The assay had a detection limit similar to that of qPCR. The specificity of the assays was evaluated using excessive amounts of mouse DNA and this did not affect the reaction. The assays were also evaluated using extracted DNA from human patient samples, demonstrating around 80% sensitivity and 100% specificity using limited clinical samples. Finally, the ease with which the cross-contamination-proof lateral flow cassette can be used to detect multiple amplicons from various nucleic acid amplification methods makes it promising for wide-ranging use in the field.

## Supporting Information

S1 Checklist STARD checklist. (DOC)

S1 Fig Sequence alignment of RPA primer targeted region of the 47 kDa gene from 8 strains of *O*. *tsutsugamushi*. The sequences of RPA primers targeted region of 47 kDa gene from 8 strains were analyzed using ClustalW2 (http://www.ebi.ac.uk/Tools/msa/clustalw2/). The bases that showed mismatch are highlighted in yellow. There are 13 bases that were mismatched out of 156 bases among the 8 strains. This is greater than 90% (91.7%) identical in sequence among these 8 strains.
(TIF)

S2 Fig The primer and probe set used for *R*. *typhi* DNA detection in RPA-nfo could not detect *R*. *conorii* and *R*. *rickettsii* DNA with similar detection limit. The RPA-nfo was performed as described in the Materials and Methods using DNA extracted from *R*. *typhi*, *R*. *conorii* and *R*. *rickettsii* with different copy number as determined by qPCR. Lane N: negative, lanes 1–4 contained 425, 170, 85 and 40 copies/reaction of *R*. *typhi* DNA, respectively, lane 5 contained 10^4^ *R*. *rickettsii* DNA, and lanes 6–7 contained 10^4^ and 10^3^ copies/reaction of *R*. *conorii* DNA.
(TIF)
