# Supplementary figures and images for: Development of Recombinase Polymerase Amplification Assays for Detection of Orientia tsutsugamushi or Rickettsia typhi
Source: PLoS Negl Trop Dis. 2015 Jul 10;9(7):e0003884. doi: 10.1371/journal.pntd.0003884 (PMC4498641; doi:10.1371/journal.pntd.0003884)

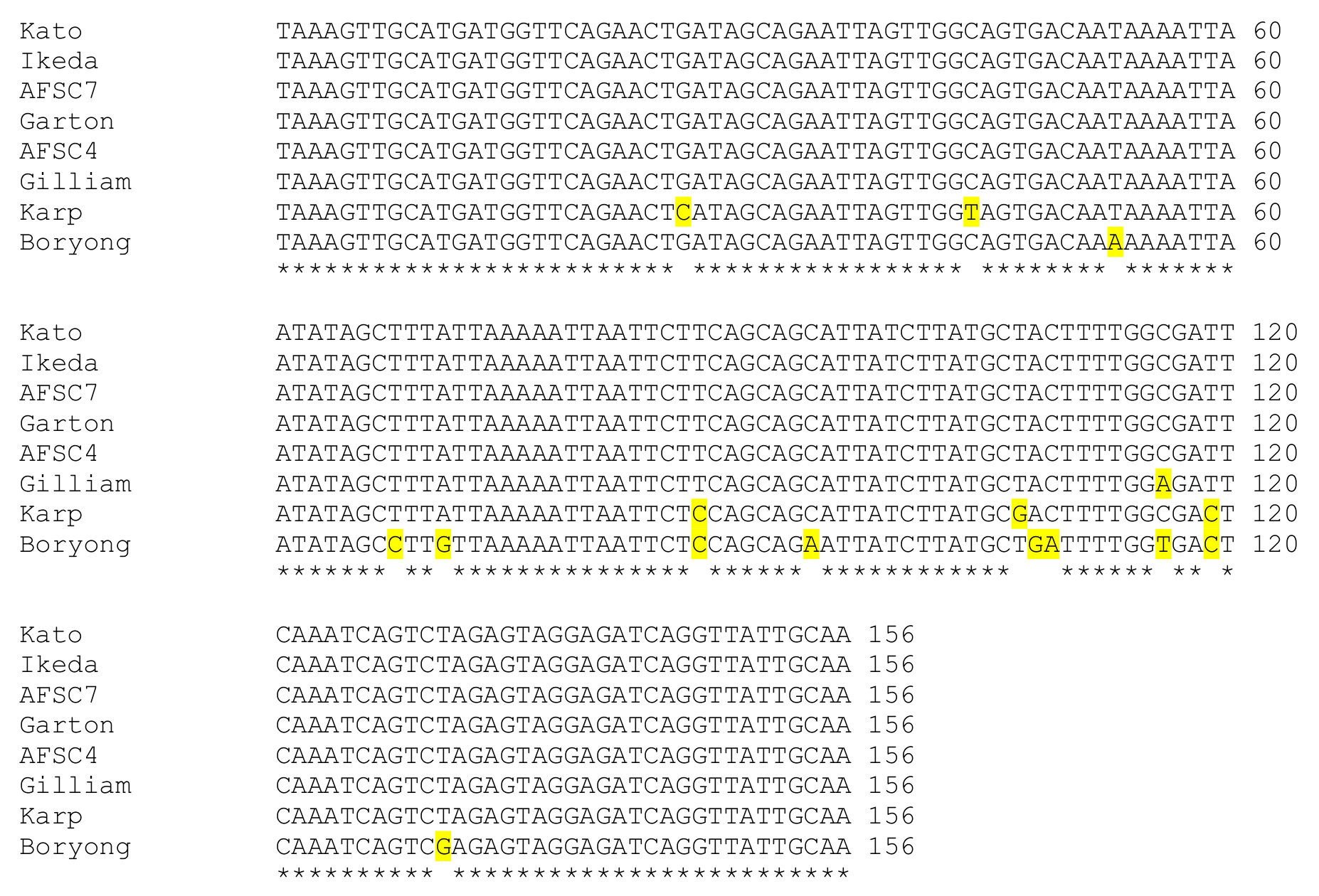

Supplement: S1 Fig — The sequences of RPA primers targeted region of 47 kDa gene from 8 strains were analyzed using ClustalW2 (http://www.ebi.ac.uk/Tools/msa/clustalw2/). The bases that showed mismatch are highlighted in yellow. There are 13 bases that were mismatched out of 156 bases among the 8 strains. This is greater than 90% (91.7%) identical in sequence among these 8 strains. (TIF) [file pntd.0003884.s002.tif]

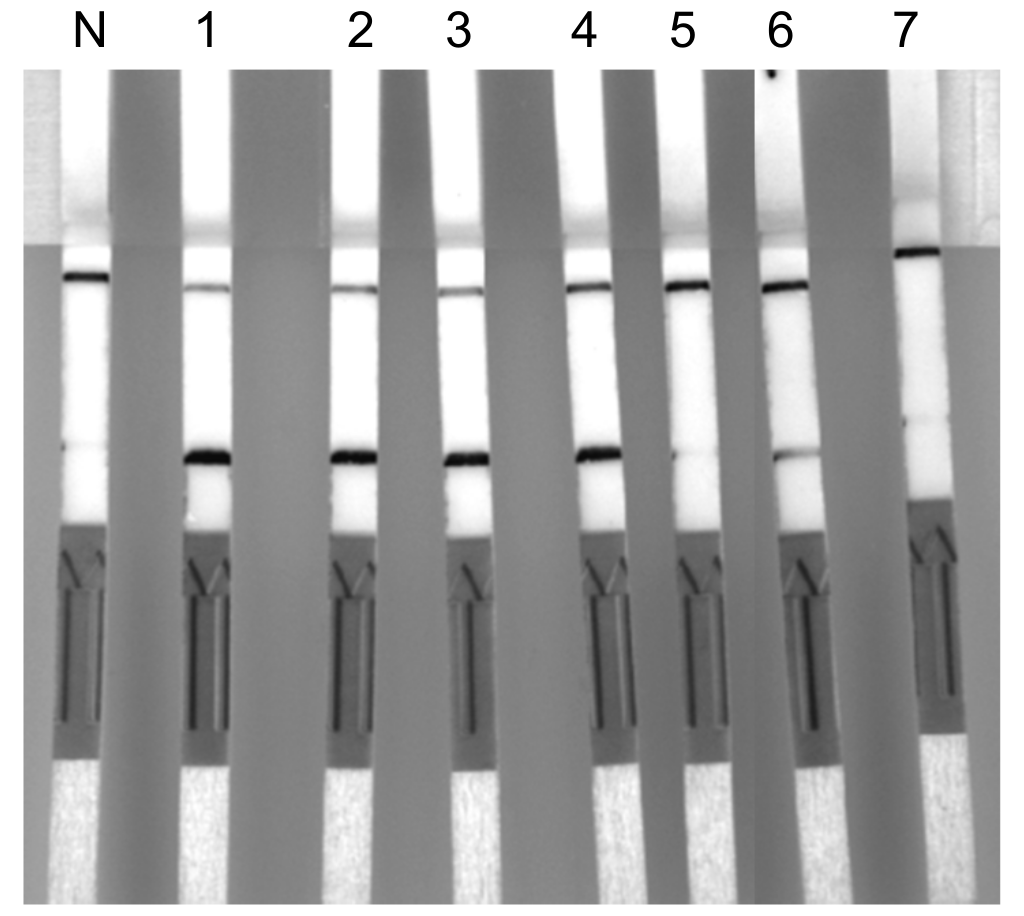

Supplement: S2 Fig — The RPA-nfo was performed as described in the Materials and Methods using DNA extracted from R. typhi, R. conorii and R. rickettsii with different copy number as determined by qPCR. Lane N: negative, lanes 1–4 contained 425, 170, 85 and 40 copies/reaction of R. typhi DNA, respectively, lane 5 contained 104 R. rickettsii DNA, and lanes 6–7 contained 104 and 103 copies/reaction of R. conorii DNA. (TIF) [file pntd.0003884.s003.tif]
